# CircSorbs1 regulates myocardial regeneration and reduces cancer therapy-related cardiovascular toxicity through the Mir-99/GATA4 pathway

**DOI:** 10.1007/s12672-024-01075-0

**Published:** 2024-07-30

**Authors:** Kang Huang, Denggao Huang, Qiang Li, Jiangting Zeng, Ting Qin, Jianghua Zhong, Zanrui Zhong, Shijuan Lu

**Affiliations:** 1 Department of Cardiology, Central South University Xiangya School of Medicine Affiliated Haikou Hospital, No.43, Renmin Avenue, Haikou, 570208 Hainan China; 2 Central Laboratory, Central South University Xiangya School of Medicine Affiliated Haikou Hospital, Haikou, 570208 Hainan China

**Keywords:** CircSorbs1, Mir-99, Myocardial regeneration, Cancer therapy-related cardiovascular toxicity

## Abstract

Due to the cancer therapy-related cardiovascular toxicity, heart failure following cancer therapy has a significant mortality rate. Gene-targeted therapy promotes the re-entry of existing cardiomyocytes into the cell cycle to achieve myocardial regeneration, which is a promising strategy for preventing and treating heart failure after myocardial infarction. Circular RNAs (circRNAs) are considered as potential targets for myocardial regeneration due to their strong stability, resistance to degradation, and potential role in heart development and cardiovascular diseases. By comparing the myocardial tissue of mice in the sham operation group and the Doxorubicin therapy group (DOX), we observed a significant decrease in Cirsorbs expression in the DOX group. Cirsorbs was predominantly localized in cardiomyocytes and exhibited high conservation. Subsequent investigations revealed that Cirsorbs could promote myocardial proliferation and inhibit myocardial apoptosis. Mechanistic studies further demonstrated that Cirsorbs could bind to miR99 and reduce its expression level. Meanwhile, miR99 was found to bind to GATA4 mRNA and decrease its expression level. The binding of Cirsorbs to miR99 alleviated the repression of miR99, thereby enhancing GATA4 expression and the transcription of downstream cyclin A2 and cyclin E1. This, in turn, increased cardiomyocyte proliferation and reduced apoptosis. In conclusion, Cirsorbs holds promise as an effective target for myocardial regeneration in reducing cancer therapy-related cardiovascular toxicity.

**Supplementary Information:**

The online version contains supplementary material available at 10.1007/s12672-024-01075-0.

## Introduction

Myocardial infarction is the leading cause of end-stage heart failure. While drug intervention and coronary intervention have enabled patients with myocardial infarction to receive effective reperfusion therapy in a timely manner, these methods do not address the loss of necrotic cardiomyocytes at the “source,” resulting in high mortality rates among patients with end-stage heart failure following myocardial infarction [1–3]. The reduction in cardiomyocyte numbers due to irreversible necrosis and apoptosis is the underlying cause of heart failure after myocardial infarction. Therefore, myocardial regeneration to enhance repair after myocardial infarction represents the fundamental approach for preventing and treating heart failure subsequent to myocardial infarction.

Previously, it was believed that the adult mammalian heart was a terminally differentiated organ that permanently exited the cell cycle and lost its regenerative capacity after reaching adulthood [4]. However, recent studies have challenged this notion by demonstrating the strong regenerative capacity of the heart in lower vertebrates and newborn mammals [5, 6]. Further research has revealed that adult cardiomyocytes also possess the potential for proliferation and self-renewal, albeit at a low rate [7]. To increase the number of cardiomyocytes, there are four major approaches that have been explored: exogenous stem cell transplantation, cellular reprogramming, tissue engineering, and endogenous myocardial regeneration [8, 9]. These approaches aim to either introduce new cardiomyocytes into the heart or stimulate the existing cardiomyocytes to proliferate and regenerate.

Gene targeted therapy for promoting endogenous myocardial regeneration involves the manipulation of target genes to regulate the expression of specific proteins, thereby stimulating quiescent cardiomyocytes to re-enter the cell cycle and promote cardiomyocyte proliferation for the repair of damaged myocardium. Previous studies have shown that the overexpression of cyclin D2 and cyclin A2 in the hearts of rats with myocardial infarction can induce cardiomyocyte proliferation and facilitate the repair of scarred areas following myocardial infarction [10, 11].

Furthermore, other studies have identified 40 miRNAs that effectively increase cardiomyocyte division in neonatal rats. Notable examples include miR-199a, miR-99p, and miR-128, which have been shown to promote cardiomyocyte re-entry into the cell cycle and facilitate myocardial regeneration in both neonatal and adult rats [12–14]. These findings highlight the potential of miRNAs as therapeutic targets for promoting endogenous myocardial regeneration.

Circular RNA (circRNA) is a type of non-coding RNA molecule that forms a closed loop structure without a 5' cap structure and a 3’ poly(A) tail. It is primarily located in the cytoplasm or stored in exosomes [15]. Compared to traditional linear RNA, circRNA is more stable and resistant to degradation as it is not affected by RNA exonucleases. It has been found to be widely present in various eukaryotes. circRNAs play important roles in cardiac development and the progression of cardiovascular diseases. For example, circ-Amotl1 has been shown to promote AKT protein phosphorylation and nuclear translocation, thereby reducing myocardial apoptosis and ventricular remodeling after myocardial infarction [16]. Another circRNA, circSlc8a1, can affect the expression of serum response factor Srf, connective tissue growth factor Ctgf, and adrenergic receptor Adrb1 by sequestering mir-133, thereby alleviating cardiac hypertrophy [17]. These findings highlight the significant impact of circRNAs on heart development and the prevention and treatment of cardiovascular diseases. They also suggest that circRNA may serve as a novel target for gene therapy in myocardial regeneration following myocardial infarction.

The main mechanism of circRNAs involves their function as endogenous competing RNAs (ceRNAs). As ceRNAs, circRNAs regulate gene expression through a common mode of gene regulation. Specifically, circRNAs can act as ceRNAs by competing with microRNA response elements to regulate the expression level of microRNAs, thereby influencing cellular functions. In our previous study, we discovered that circSorbs1 can bind to two isoforms of mmu-miR-99, namely mmu-miR-99a-3p and mmu-miR-99b-3p. It has been shown that miR-99 can induce proliferation in dedifferentiated cardiomyocytes by regulating Gata4, a transcription factor associated with myocardial regeneration [14]. Gata4 primarily functions by promoting the expression of cell cycle factors, such as cyclin A2 and cyclin E1 [18, 19]. Based on these findings, we speculate that circSorbs1 acts as a ceRNA by sequestering miR-99, thereby influencing the Gata4/cyclin signaling axis and mediating myocardial regeneration.

## Methods

### Animals and diets

The normal chow diet (containing 0.2% total choline, 33.58% corn, 25% flour, 17.5% soybean, 13.3% wheat flour, 4.2% fish meal, 2.5% soybean oil, 2% dicalcium phosphate, 1.3% calcium carbonate, 0.3% salt, 0.08% minerals, and 0.04% vitamin) was acquired from Guangdong Medical Laboratory Animal Center (Guangdong, China). C57BL/6 J mice were purchased from Vitalriver Biotech Co., Ltd. (Beijing, China). Animal protocols were approved by the Animal Experiment Committee of Central South University Xiangya School of Medicine Affiliated Haikou Hospital (Haikou, China). The animals used in the study were 8-week-old male mice.

### RNA isolation, reverse transcription, and quantitative real-time PCR

Total RNA was extracted from cardiac muscle cells with TRIzol reagent. Then, cDNA synthesis was performed via reverse transcription using a 10 µl system that was prepared using 5 × Evo M-MLVRT Master Mix. Quantitative real-time PCR was performed using 2 × SYBR Green Pro Taq HS Premix (Takara). The results were normalized based on the expression of the housekeeping gene β-actin. The quantitative results were determined using the ΔΔCT method. The primers used in this study are described in the Supplementary Material.

Primer sequence:

GATA Primers:

Forward primer: CCTCCATCCATCCAGTGCTGTC; Reverse primer: ACCAGGCTGTTCCAAGAGTCC.

Cyclin A2 Primers:

Forward primer: TTGTGGACTGGCTGGTTGAGG; Reverse primer: ACACAGACATGGAGGAGAGGAATC.

Cyclin E1 Primers:

Forward primer: GTCCTGGATGTTGGCTGCTTAG; Reverse primer: CTCTATGTCGCACCACTGATAACC.

miR-99b-3p Primers:

Forward primer: GCGCAAGCTCGTGTCTGTG; Reverse primer: AGTGCAGGGTCCGAGGTATT.

### Western blotting

Cardiac muscle cells were lysed with a mixture of RIPA and protease inhibitors. The protein concentrations were determined using the BCA method. Proteins were separated by SDS‒PAGE and transferred to PVDF membranes. The blots were then blocked and incubated with specific primary antibodies and secondary antibodies. Target proteins were detected with a Kodak Image Station 2000MM imaging system using chemiluminescence (ECL) reagents. The primary antibodies included an anti-GATA4 antibody (1:1000, Abcam), anti-Cyclin A2 antibody (1:1000, Abcam), anti-Cyclin E1 antibody (1:1000, Abcam), and anti-β-actin antibodies (1:1000, Fude). Secondary antibodies were purchased from Hangzhou Fude Co., Ltd.

### Fluorescence in situ hybridization (FISH)

Cy3-labeled circSorbs1 (5′ CY3-AGCAGGTCTCATCTGTGGGC-3′) was used to detect the cellular localization of circSorbs1. FISH analysis was performed using a Fluorescent In Situ Hybridization Kit (Invritrogen, California, USA) according to the manufacturer’s instructions. Nuclear were stained with DAPI dye (Invritrogen, California, USA). The images were photographed under the Olympus fluorescence microscope (Olympus, Tokyo, Japan).

### Dual-luciferase reporter assay

The sequences of circSorbs1 containing the WT or Mut binding site of miR-99 were cloned and inserted into the pGL3-basic vectors (Promege, Madison, USA). In 12-well plates, 293 T cells were cultured to approximately 70% confluence and then co-transfected with either WT or Mut luciferase reporter vector (1 μg) and either mimic miRNAs or negative control (NC) (1 μg). After 48 h of incubation, the activities of firefly and Renilla luciferase were measured using the Dual Luciferase Reporter Assay Kit (Promega, Madison, USA).

### Cell cycle and apoptosis assays

For cell cycle, cells were harvested and fixed in pre-cold 70% ethanol at 4 °C overnight, then stained with propidium iodide (PI) and measured by the flow cytometry (BD, NY, USA). The cells were stained with Annexin V-FITC and PI and was measured by flow cytometry according to the manufacturer’s instructions (C1062S, Beyotime, China).

### Statistical analyses

All the data are presented as the mean ± SD. GraphPad 8.0.1 was used for the statistical analysis of the data. Two groups of independent samples were compared using Student’s t test, and three or more groups of independent samples were compared using one-way ANOVA. A P value (two-tailed) < 0.05 was considered statistically significant.

## Result

### Expression of CircSorbs1

**Fig. 1:**
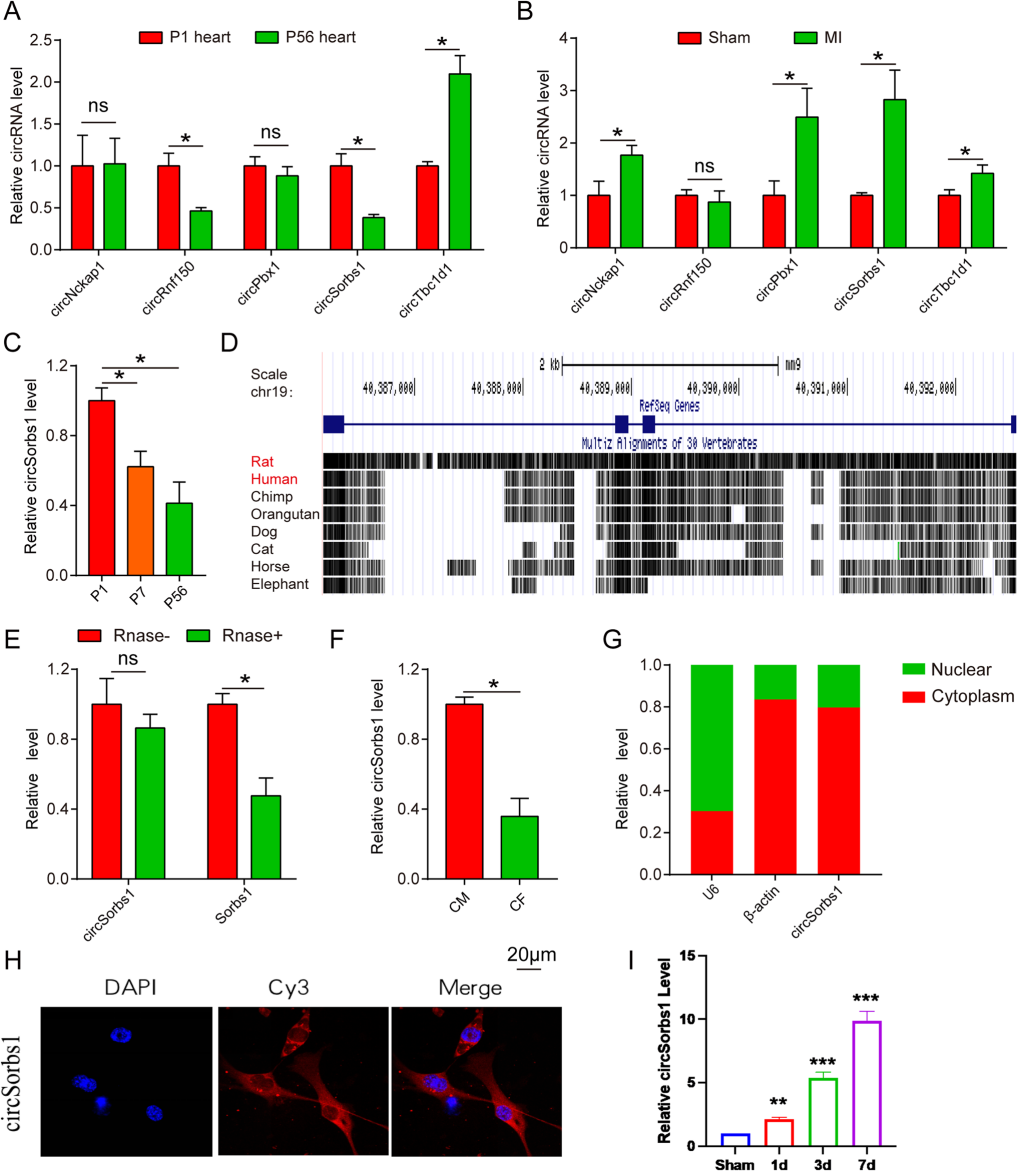
The expression of circSorbs1. **A** Expression levels of different circRNAs in neonatal and adult mice were assessed by RT-qPCR. (*p < 0.05, n = 3). **B** Expression levels of different circRNAs in the heart of sham group and MI group were assessed by RT-qPCR. (*p < 0.05, n = 3). **C** Expression levels of circSorbs1 in the hearts of mice of different ages were assessed by RT-qPCR. (*p < 0.05, n = 3). **D** UCSC database revealed the conservation of circSorbs. **E**, Expression levels of circSorbs1 in cardiomyocytes of control group and ribonuclease treatment group were assessed by RT-qPCR. (*p < 0.05, n = 3). **F** Expression levels of circSorbs1 in cardiomyocytes and fibroblasts were assessed by RT-qPCR. **G** The distribution of circSorbs1 in cardiomyocytes. (*p < 0.05, n = 3). **H** Cellular localization of circSorbs1 was detected by FISH. (n = 3). **I** Expression levels of circSorbs1 in cardiac tissues at different time after myocardial infarction by RT-qPCR. (**p < 0.01, ***p < 0.005, n = 3)

In our study, we performed RT-qPCR to assess the expression of CircSorbs1 in cardiomyocytes isolated from neonatal mice and 7-week-old mice. The results showed a significant decrease in CircSorbs1 expression in cardiomyocytes from 7-week-old mice compared to neonatal mice (Fig. 1A, C). Furthermore, we observed an increase in CircSorbs1 expression in the myocardial cells of the MI group compared to the control group (Fig. 1B). In addition, the expression level of CirSorbs1 in cardiac tissue gradually increased with the increase of myocardial infarction time (Fig. 1I). These findings suggest that CircSorbs1 may be involved in cardiomyocyte proliferation. To investigate the conservation of CircSorbs1, we utilized the UCSC database and found that it is conserved in mice, rats, and humans (Fig. 1D). Additionally, we conducted ribonuclease treatment on cardiomyocytes and observed that the level of CircSorbs1 was not significantly altered, indicating that it is not a linear RNA (Fig. 1E). Moreover, we observed that CircSorbs1 was predominantly located in cardiomyocytes compared to fibroblasts (Fig. 1F). Overall, our results indicate that CircSorbs1 is a well-conserved circular RNA primarily located in the nucleus of cardiomyocytes (Fig. 1G, H). We propose that CircSorbs1, as a circular RNA mainly located in the nucleus of cardiomyocytes, is associated with cardiomyocyte proliferation.

### CircSorbs1 promoted the proliferation and division of cardiomyocytes

**Fig. 2:**
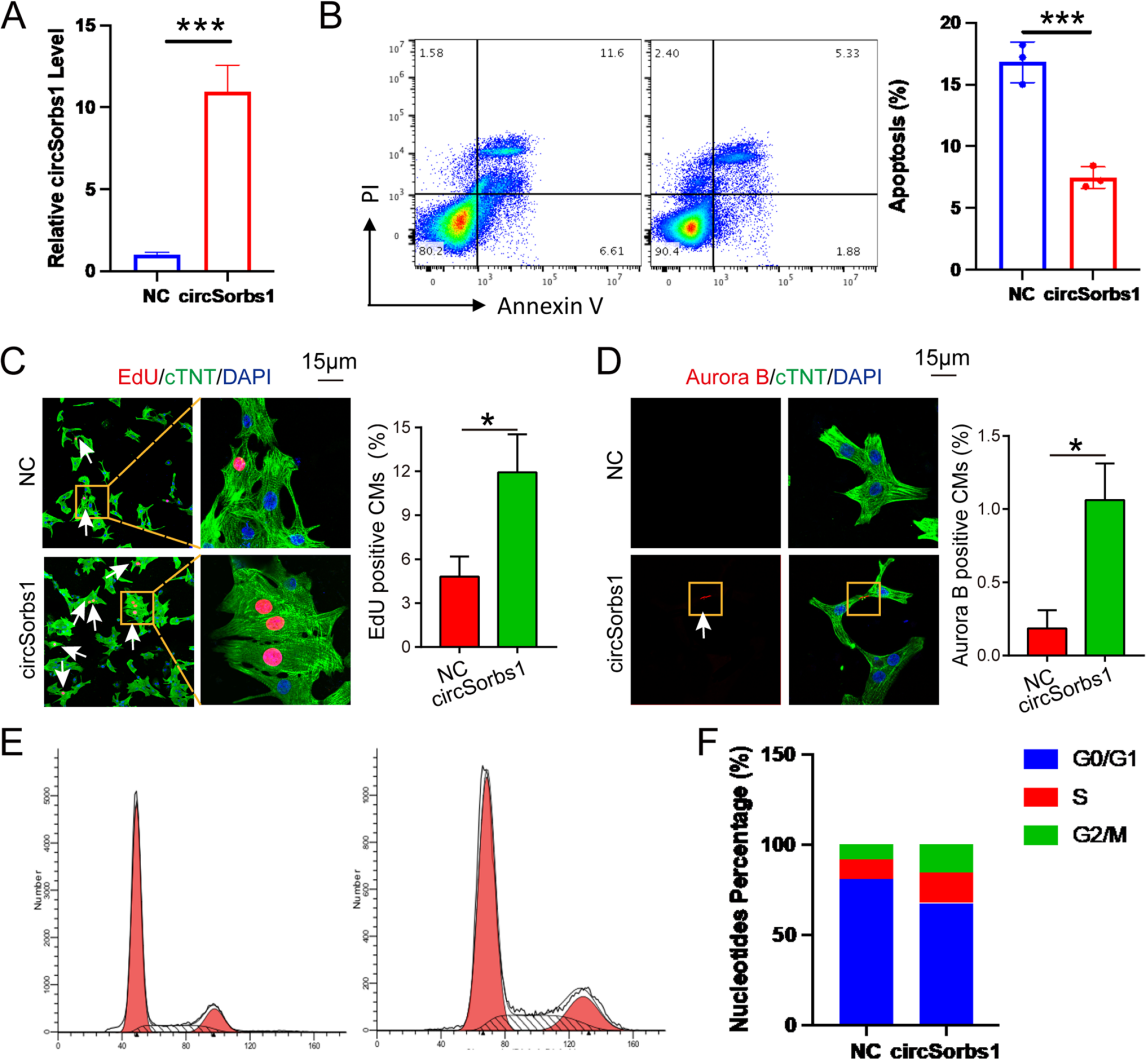
The role of CircSorbs1 in the proliferation and division of cardiomyocytes. **A** Expression levels of circSorbs1 was assessed by RT-qPCR. (***p < 0.005, n = 3). **B** Level of apoptosiscontrol was assessed by flow cytometry. (***p < 0.005, n = 3). **C** Proportion of Edu-positive cells after circSorbs1 overexpression. (*p < 0.05, n = 3). **D** Proportion of Aurora B-positive cells after circSorbs1 overexpression. (*p < 0.05, n = 3). **E**, **F** Alterations of cell cycle was assessed by flow cytometry

Through our experiments, we have confirmed that CircSorbs1 can indeed promote the proliferation and division of cardiomyocytes. We constructed plasmids to overexpress CircSorbs1, which successfully increased the level of CircSorbs1 in cardiomyocytes (Fig. 2A). Flow cytometry analysis revealed that overexpression of CircSorbs1 led to a decrease in cardiomyocyte apoptosis (Fig. 2B) and an increase in Edu-positive cells, indicating enhanced cardiomyocyte proliferation (Fig. 2C). Additionally, the proportion of Aurora B-positive cells, which is indicative of cardiomyocyte division, also increased (Fig. 2D). Furthermore, we examined cell cycle alterations using flow cytometry and observed that more primary cardiomyocytes accumulated in the synthetic and gap 2 phases of the cell cycle (Fig. 2E, F). These findings provide strong evidence that CircSorbs1 plays a role in promoting the proliferation and division of cardiomyocytes.

### CircSorbs1 reduces miR-99 in cardiomyocytes by acting as a sponge for the miR-99

**Fig. 3:**
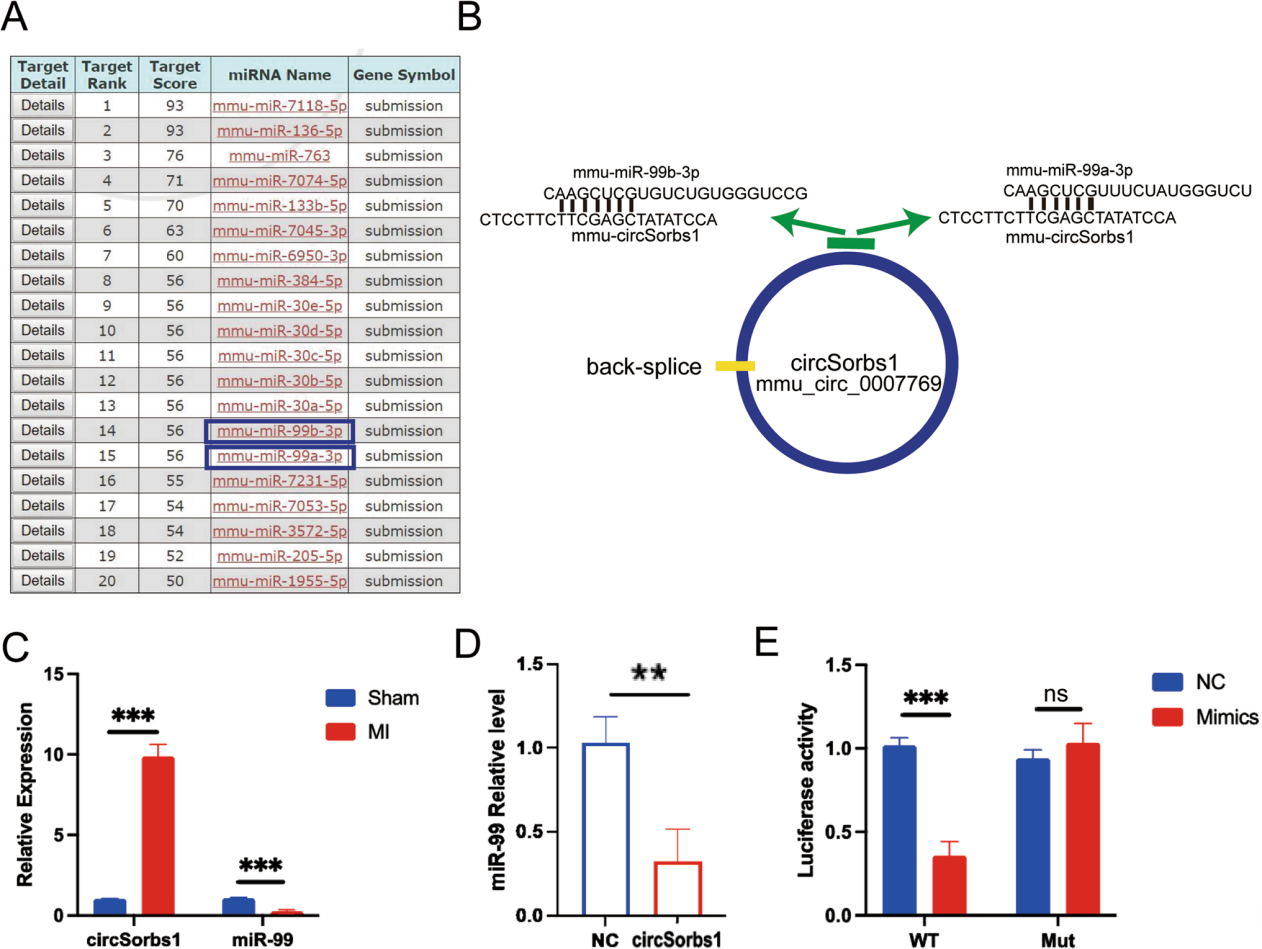
**A**, **B** Binding sites of circSorbs1 with mmu-miR-99a-3p and mmu-miR-99b-3p were analyzed by bioinformatics. **C** Expression levels of circSorbs1 and miR-99 in the heart of sham operation group and MI group were assessed by qPCR. (***p < 0.005, n = 3). **D** Expression level of miR-99 in cardiomyocytes overexpressing circSorbs1 was assessed by qPCR. (**p < 0.01, n = 3). **E** Luciferase activity of wild-type circSorbs1 and circSorbs1 with miR-99 binding site mutants was assessed by luciferase reporter assay. (***p < 0.005, n = 3)

To further investigate the mechanism by which CircSorbs1 promotes the proliferation and division of cardiomyocytes, we conducted additional research. Through bioinformatics prediction analysis, we discovered that CircSorbs1 binds to miR99 (Fig. 3A, B), suggesting that CircSorbs1 may act as a sponge to bind miR99 and reduce its level in cardiomyocytes. Furthermore, we compared the expression levels of circSorbs1 and miR99 between the sham group and the myocardial infarction (MI) group (Fig. 3C). The results demonstrated that circSorbs1 was up-regulated while miR99 was down-regulated in the MI group, indicating a negative correlation between the expression levels of circSorbs1 and miR99. This suggests that CircSorbs1 is capable of inhibiting the level of miR99. To validate this conclusion, we overexpressed CircSorbs1 in cardiomyocytes and observed a decrease in the expression of intracellular miR99 compared to the control group, as confirmed by RT-qPCR (Fig. 3D). This provides further evidence that CircSorbs1 can reduce the level of miR99 in cardiomyocytes. To confirm the specificity of the binding between CircSorbs1 and miR99, we constructed CircSorbs1 plasmids with mutations in the miR99-binding site. Luciferase reporter assays revealed that the luciferase activity of the circSorbs1 mutant was not significantly altered, indicating that the binding of CircSorbs1 to miR99 is specific (Fig. 3E). In summary, these findings demonstrate that CircSorbs1 specifically binds to miR99 and acts as a sponge to reduce its level in cardiomyocytes. Previous studies have shown that miR99 can influence the proliferation of zebrafish cardiomyocytes. Therefore, it is plausible that the promotion of cardiomyocyte proliferation and division by CircSorbs1 is related to its ability to reduce the level of miR99 in cardiomyocytes.

### CircSorbs1 regulates Gata4/Cyclin signaling axis to mediate myocardial regeneration by adsorb miR-99

**Fig. 4:**
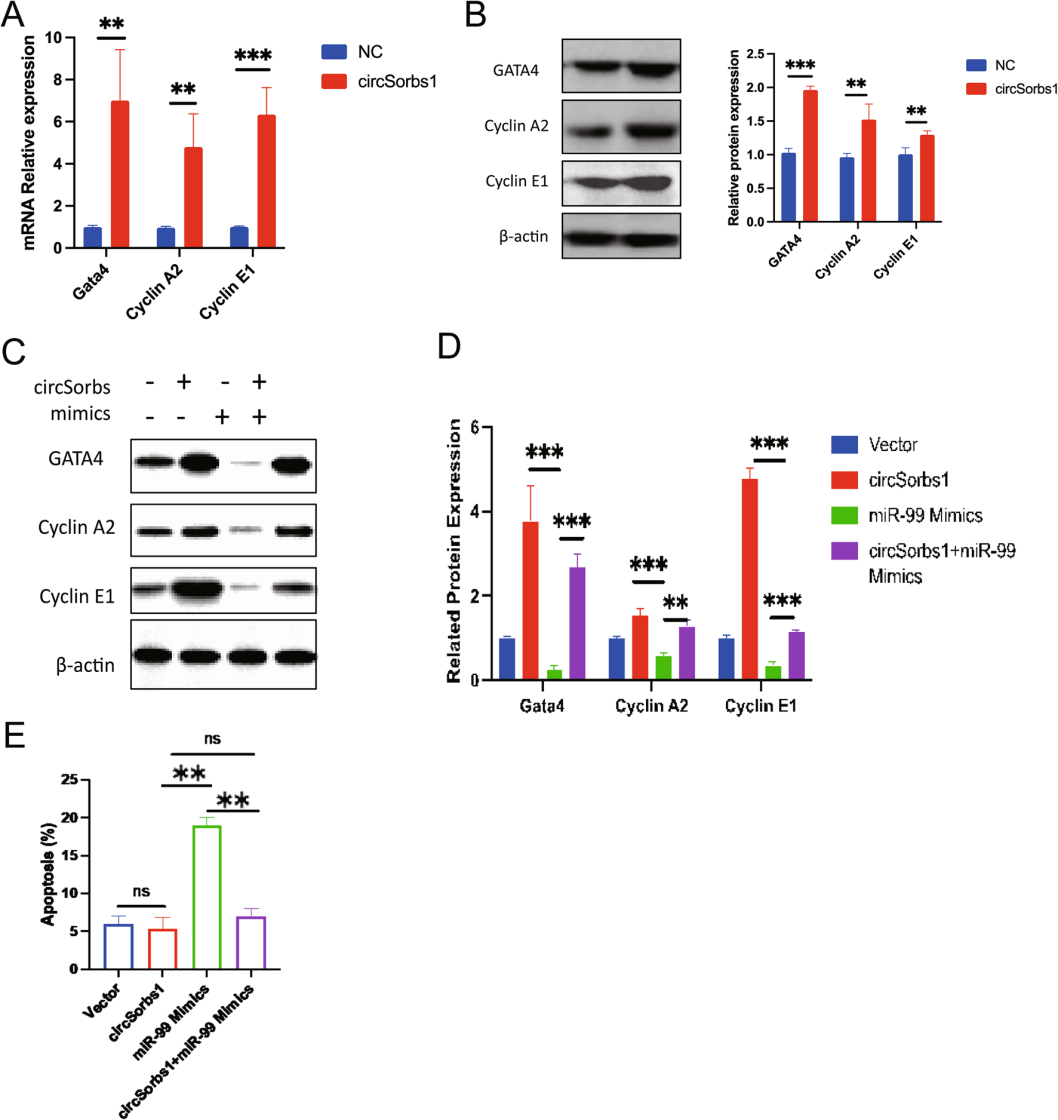
Cardiomyocytes of the control group and the circSorbs1 overexpression group. **A** RNA expression levels of Gata4, cyclin A2, and cyclin E1 were assessed by qPCR. (**p < 0.01, ***p < 0.005, n = 3). **B** Protein expression levels of Gata4, cyclin A2 and cyclin E1 were assessed by Western blotting. (**p < 0.01, ***p < 0.005, n = 3). **C**, **D** Effect of miR99 on cyclin A2 and cyclin E1 after CircSorbs1 overexpression by RT-qPCR. (**p < 0.01, ***p < 0.005, n = 3). **E** Effect of miR99 on apoptosis and cell cycle after CircSorbs1 overexpression was detected by flow cytometry. (**p < 0.01)

To investigate the potential relationship between CircSorbs1 and miR99 in promoting the proliferation and division of cardiomyocytes, we conducted a comprehensive study. Previous research has indicated that GATA4 plays a crucial role in promoting the expression of cell cycle factors, such as CyclinA2 and CyclinE1 [18, 19]. Therefore, we aimed to determine whether CircSorbs1 affects cardiomyocyte proliferation by influencing GATA4. Following the overexpression of CircSorbs1 in cardiomyocytes, we observed an increase in the mRNA and protein expression levels of Gata4, cyclin A2, and cyclin E1, as confirmed by RT-qPCR and WB analysis (Fig. 4A, B). Moreover, after the overexpression of CircSorbs1 was combined with the addition of miR-99 simulators, we discovered that miR99 eliminated the effects of CircSorbs1 overexpression on cyclin A2 and cyclin E1 (Fig. 4C, D). In addition, flow cytometry also confirmed that miR99 eliminated the inhibition of CircSorbs1 overexpression on apoptosis and its effect on cell cycle (Fig. 4E). Through the above studies, we confirmed that CircSorbs1 adsorbs miR99 by CeRNA mechanism to increase the expression of GATA4, thereby increasing the proliferation and division of cardiomyocytes and reducing their apoptosis.

### CircSorbs1 is associated with Doxorubicin induced myocardial injury

**Fig. 5:**
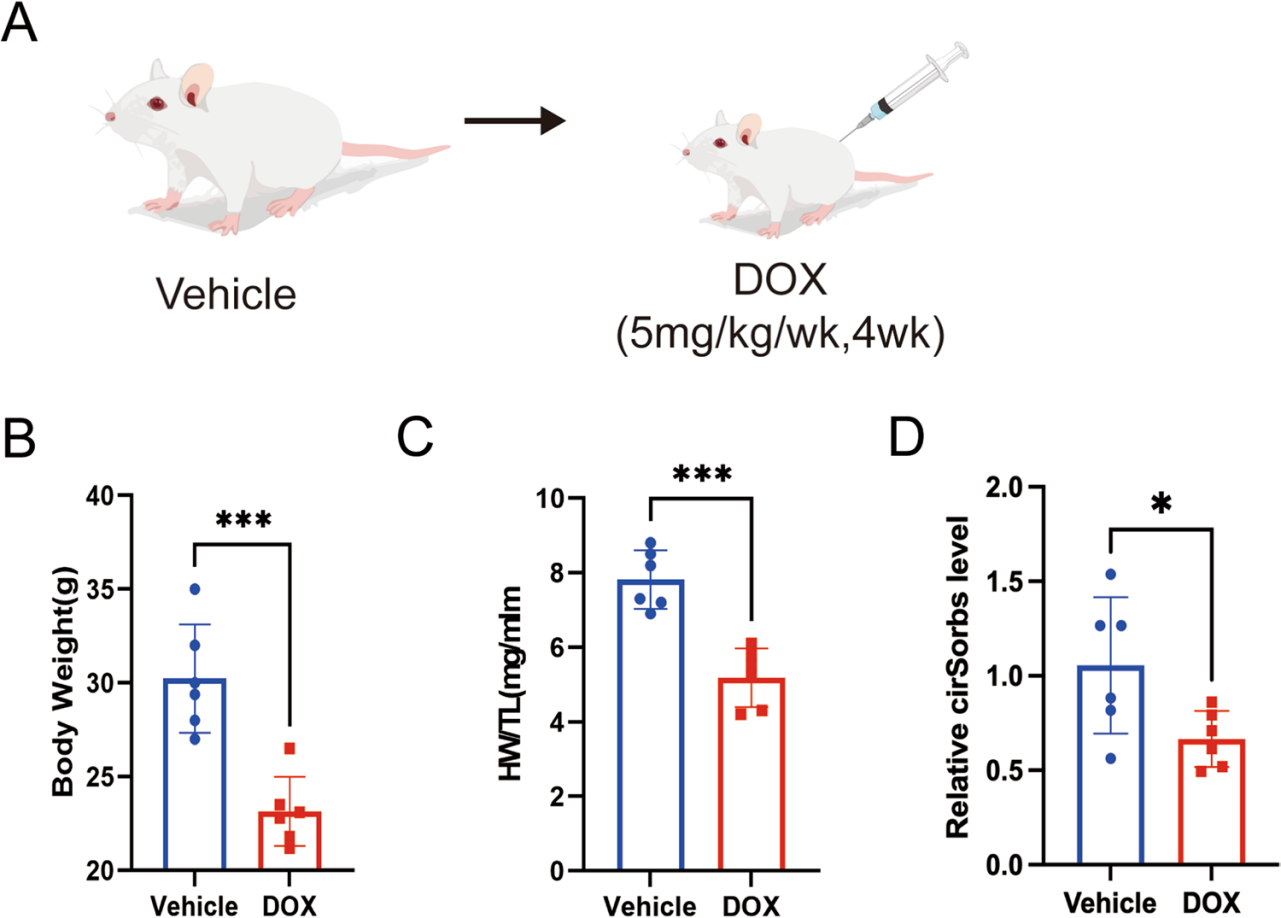
CirSorbs is associated with Doxorubicin induced myocardial injury. A.Experiment design. A chronic DoIC mouse model was established in C57BL/6 J mice,All tests and analysis were implemented 2 weeks after the last DOX injection. B.Body weight of mice (n = 6). **C**.Ratio of heart weight to tibia length (HW/TL; n = 6). **D**.The level of cirSorbs (n = 6). *p < 0.05, ***p < 0.005

Previous research suggested that the antitumor drug Doxorubicin can induce myocardial injury [20] However, its relationship with CirRNA is not clear. To this end, we constructed Doxorubicin induced myocardial injury model of mice (Fig. 5A). By observing the hearts of DOX treated mice, it was found that the hearts of DOX treated mice were smaller (Fig. 5B, C), and the level of cirSorb in the heart tissue of mice was decreased (Fig. 5D), indicating that Circsorb was related to Doxorubicin induced myocardial injury.

## Discussion

Our investigation revealed that circSorbs1, a circular RNA predominantly localized in the nucleus of cardiomyocytes, plays a crucial role in promoting cardiomyocyte proliferation and division. Through bioinformatics prediction analysis and luciferase reporter assays, we identified a specific binding interaction between circSorbs1 and miR99. Furthermore, qPCR analysis indicated that circSorbs1 reduces the expression level of miR99 in cardiomyocytes. Notably, miR99 is known to regulate the transcription factor Gata4, which is associated with myocardial regeneration [14]. Upon overexpression of circSorbs1, we observed an upregulation of Gata4, cyclin A2, and cyclin E1 expression levels. Collectively, these findings suggest that circSorbs1 modulates the expression of GATA4 by sequestering miR99, thereby regulating GATA4-mediated myocardial regeneration and facilitating post-myocardial infarction repair.

circRNAs play a crucial role in cardiac development and the pathogenesis of cardiovascular diseases. Previous studies have demonstrated the therapeutic potential of circRNAs in myocardial infarction. For instance, circ-Amotl1 has been shown to reduce myocardial apoptosis and ventricular remodeling post-myocardial infarction by promoting AKT protein phosphorylation and nuclear translocation [16]. Similarly, circ-Foxo3 has been found to delay the senescence of mouse embryonic fibroblasts by binding to and modulating the expression of the anti-aging protein ID-1 [21]. These findings highlight the potential of circRNAs as novel targets for gene therapy in myocardial regeneration following myocardial infarction. Furthermore, previous studies have established a close association between Sorbs1 and various metabolic disorders, including diabetes, obesity, and gastrointestinal cancer. Jin et al. demonstrated that Sorbs1 can be targeted by miRNAs to regulate the progression of gastric cancer [22], suggesting that circ-SORBS1 may regulate gene expression by acting as a miRNA sponge. Additionally, Zhao et al. confirmed the presence of numerous miRNA binding sites on circ-SORBS1, further supporting its potential as a miRNA sponge [23]. These findings underscore the significant potential of circ-SORBS1 as a regulator of gene expression through miRNA sequestration.

The ceRNA (competing endogenous RNA) regulatory mechanism, where circRNA acts as a ceRNA to regulate gene expression levels by competing with microRNAs through microRNA response elements, is a common pattern of gene expression regulation. This regulatory mechanism allows circRNA to influence cellular functions by modulating the availability of microRNAs. Studies have shown that intervention of differentially expressed mRNAs or miRNAs in vitro and in vivo can induce cardiomyocytes to re-enter the cell cycle and promote cardiomyocyte proliferation [24]. Previous research has identified specific circRNAs that affect cell proliferation through the ceRNA regulatory mechanism. For example, circSlc8a1 has been found to alleviate cardiac hypertrophy by sequestering mir-133 and subsequently affecting the expression of serum response factor (Srf), connective tissue growth factor (Ctgf), and adrenergic receptor (Adrb1) [17]. Similarly, circCDYL regulates cell proliferation by inhibiting the activity of miR-4793-5p through the “molecular sponge” mechanism [25]. These findings highlight the role of circRNAs as important regulators of cell proliferation through their ceRNA activity.

GATA4 is a member of the GATA family of transcription factors and plays a crucial role in cardiomyocyte development. Loss of GATA4 in early embryonic cardiomyocytes has been shown to result in reduced cardiomyocyte proliferation and myocardial hypoplasia, leading to embryonic lethality [26]. Additionally, studies have observed a significant downregulation of GATA4 expression in the mouse myocardium between postnatal day 1 and day 7, coinciding with the loss of myocardial regeneration [18, 19]. In the adult mouse heart, GATA4 is involved in promoting cardiac hypertrophy and maintaining cardiac function during pathological pressure overload [27, 28]. Previous research has indicated that GATA4 primarily influences cardiomyocyte proliferation by promoting the expression of key cell cycle regulators, such as Ccna2 (encoding cyclin A2), Ccne1 (encoding cyclin E1), and Cdk4 (encoding cyclin-dependent kinase 4). Notably, Cdk4 is a direct target of GATA4 [29]. These findings highlight the importance of GATA4 in regulating cardiomyocyte proliferation and its role in cardiac development and pathological conditions.

Despite doxorubicin can treat and possibly cure cancer, it also can cause damage to cardiac tissues and lead to a number of side effects including heart failure and myocardial infarction. The exact mechanism of doxorubicin-induced cardiotoxicity is not fully understood. Some studies show that DOX-induced cardiotoxicity is associated with reactive oxygen species production and DNA damage [30]. From the perspective of cardiomyocyte proliferation and division, this article further discussed the mechanism of antineoplastic drugs in cardiotoxicity, which has clinical guiding significance for finding new diagnostic and therapeutic targets.

Our study has certain limitations. Firstly, although we confirmed through in vitro experiments that circSorbs1 regulates myocardial regeneration via the mir-99/Gata4 pathway, further validation is required through animal experiments to strengthen these findings. Animal models will provide a more comprehensive understanding of the in vivo relevance and potential therapeutic implications of circSorbs1. Secondly, we acknowledge the absence of data regarding the expression and function of circSorbs1 in clinical patients. Obtaining clinical data on circSorbs1 expression and its functional implications in patients would enhance our understanding of its potential as a diagnostic or therapeutic target in cardiovascular diseases. These aspects will be addressed in future studies to provide a more comprehensive analysis.

In conclusion, CircSorbs1, a circular RNA predominantly localized in the nucleus of cardiomyocytes, plays a crucial role in promoting cardiomyocyte proliferation and division. By binding to miR99, CircSorbs1 alleviates the repression of miR99, leading to increased expression of GATA4 and its downstream targets, cyclin A2 and cyclin E1. This, in turn, enhances cardiomyocyte proliferation and reduces apoptosis. Overall, CircSorbs1 holds promise as a potential therapeutic target for myocardial regeneration, with potential applications in the prevention and treatment of heart failure following myocardial infarction.

### Supplementary Information

Supplementary file 1.

## Data Availability

The study has been approved by the Biomedical Ethics Committee of Haikou City People's Hospital (ZY-IRB-FOM-063) and the Finance science and technology project of hainan province (822MS200 & 822MS202), and the study was conducted in accordance with the ethical standards set out in the 1964 Declaration of Helsinki and its later amendments or provisions.

## Abbreviations

circRNAs: Circular RNAs
DOX: Doxorubicin
miR99: Micro RNA-99
circRNA: Circular RNA
ceRNA: Competing endogenous RNA
NC: Negative control
PI: Propidium iodide
MI: Myocardial infarction
Srf: Serum response factor
Ctgf: Connective tissue growth factor
Adrb1: Adrenergic receptor
Ccna2: Cyclin A2
Ccne1: Cyclin E1
Cdk4: Cyclin-dependent kinase 4
